# Lingual Thyroid with Subclinical Hypothyroidism in a Young Female

**DOI:** 10.1155/2021/6693477

**Published:** 2021-01-29

**Authors:** Subash Thapa, Prakash Khanal

**Affiliations:** ^1^Department of Radiology, Nepal Police Hospital, Kathmandu, Nepal; ^2^Department of ENT-Head and Neck Surgery, Nepal Police Hospital, Kathmandu, Nepal

## Abstract

Thyroid tissue presenting as a nodule in the base of the tongue due to the embryonic failure to descend to the anterior neck is a rare clinical entity, called lingual thyroid. Clinical presentation varies depending upon the degree of obstruction caused by an enlarged nodule or features related to thyroid function. We report a case of a 27-year-old female who presented with a foreign body sensation in the throat with mild dysphagia for 3 weeks. The patient was diagnosed as lingual thyroid with subclinical hypothyroidism based on clinical findings, imaging, and fine-needle aspiration cytology. Conservative management with hormone suppression can result in size reduction of ectopic thyroid tissue improving symptoms without surgery. Lingual thyroid with mild symptoms and subclinical hypothyroidism can be managed conservatively.

## 1. Introduction

The thyroid gland is normally located in the pretracheal region of the anterior neck inferior to the thyroid cartilage at the level of the C5-T1 vertebra [1]. The presence of thyroid tissue outside its normal anatomical position due to embryonic failure to descend is called ectopic thyroid tissue (ETT), and when present at the base of the tongue, it is called lingual thyroid (LT). The estimated prevalence of reported ectopic thyroid is 1 per 100,000–300,000 healthy individuals [2–5], which is even more high among the patients with thyroid disease (1 per 4000 to 8000 cases) [3]. The exact prevalence of lingual thyroid is unknown since many asymptomatic patients never come to medical attention; however, a study among 200 consecutive routine necropsies on Caucasian individuals suggested that 10% of adults may harbor asymptomatic thyroid tissue along the path of the thyroglossal duct [3]. Among the patients with thyroid disease, the overall prevalence of lingual thyroid is 1 in 3000 cases [5, 6]. Lingual thyroid is common among females (70–80%) [2, 5, 6]. Most cases of LT are asymptomatic [4–9] unless an increase in gland size occurs resulting in dysphagia, dysphonia, foreign body sensation in the throat, cough, pain, bleeding, and dyspnea [2]. Meticulous diagnostic workup including clinical examination, biochemical profile, imaging tools, radioisotope scanning, and histopathology is necessary to plan the management in lingual thyroid [7, 8, 10]. The approach of size reduction by hormone suppression therapy among mild symptomatic patients with lingual thyroid can resolve symptoms to avoid surgical intervention [4, 7, 10].

## 2. Case Report

**Figure 1:**
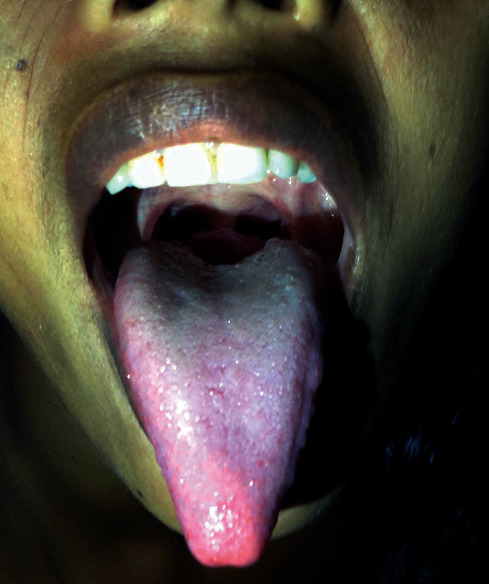
Throat examination showing midline nodule in the base of the tongue.

**Figure 2:**
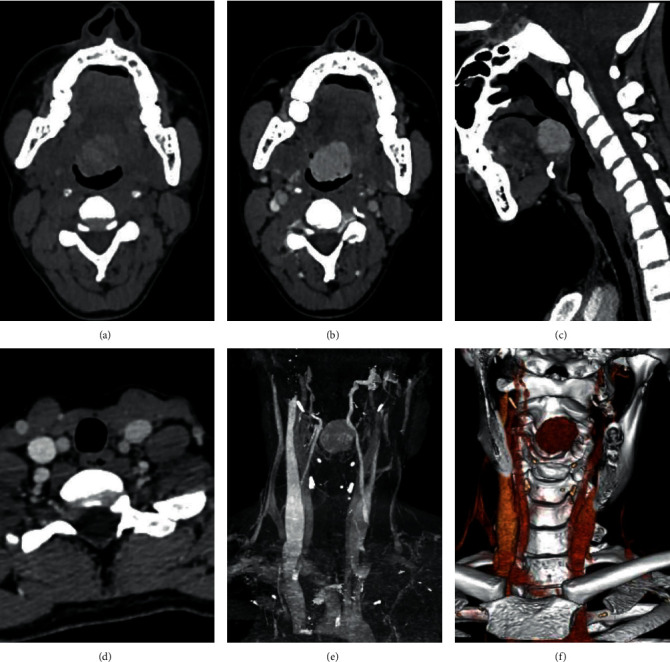
Computed tomography (CT) scan of the neck. Axial noncontrast CT image with a well-defined relatively hyperdense nodule in the midline at the base of the tongue (a), axial contrast-enhanced CT showing homogeneous enhancement (b), sagittal contrast-enhanced CT image with an enhancing nodule at the level of C2-C3 vertebra causing narrowing of the oropharyngeal lumen (c), axial contrast-enhanced CT image below the cricoid cartilage without orthotopic thyroid (d), coronal maximum intensity projection (MIP) image with a nodule in the midline (e) with absent orthotopic thyroid, and volume rendered image with a nodule in the midline (f).

**Figure 3:**
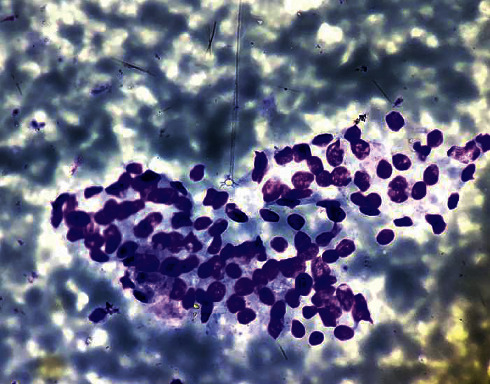
Fine-needle aspiration cytology from the nodule showing the cluster of benign-looking thyroid epithelial cells in the background of RBCs (Giemsa stain, 400x).

A 27-year-old female presented to the outpatient department with a history of foreign body sensation in the throat with mild dysphagia for 3 weeks. There was no significant medical or surgical history in the past. Throat examination revealed a well-defined nodule in the midline at the base of the tongue with intact smooth mucosa (Figure 1). The thyroid gland could not be appreciated on neck palpation. Chest X-ray was normal. Ultrasonography (USG) neck revealed the absence of a thyroid gland in its orthotopic location. No enlarged cervical lymph nodes were seen. A computed tomography (CT) scan showed a well-defined relatively hyperdense homogeneous enhancing nodule measuring 24 × 24 × 22 mm in the midline at the base of the tongue without calcification. No thyroid gland was seen in the orthotopic location (Figure 2). Thyroid function tests showed normal FT3: 3.3 pg/mL (reference value 1.2–4.1 pg/mL), normal FT4: 13.1 pg/mL (reference value 8.9–17.1 pg/mL), and slightly elevated TSH: 5.7 mIU/mL (reference value 0.3–4.5 mIU/mL). Fine-needle aspiration cytology (FNAC) from the nodule at the base of the tongue showed a cluster of benign-looking thyroid epithelial cells in the background of RBCs (Figure 3). Thyroid scintigraphy with technetium (Tc-99 m) showed uptake of the radiotracer at the region of the base of the tongue. No uptake was seen in the normal thyroid gland location.

Based on the clinical examination, thyroid function test report, USG, and CT findings, a definitive diagnosis of lingual thyroid with subclinical hypothyroidism was made, and the patient was planned for conservative pharmacological management with levothyroxine supplement. Symptoms gradually resolved by 2 months. The patient is now on a regular follow-up.

## 3. Discussion

The thyroid gland is the first endocrine gland to develop beginning around 20–24 days of gestation [1] from the endoderm of the first and second pharyngeal pouch. It descends anteroinferiorly to reach the pretracheal region inferior to the thyroid cartilage at the level of the C5-T1 vertebra by the 7^th^ week [1, 3, 7]. During its migration, the thyroid gland is attached to the base of the tongue by the thyroglossal duct which solidifies and gradually degenerates by the 10^th^ week of gestation [1]. Failure to descend to the normal anatomical position results in the ectopic location of the thyroid tissue. A few authors state that maternal antithyroid immunoglobulins might be responsible for impaired descend during early life [5, 6, 9]. The commonest location of the ectopic thyroid tissue (ETT) in the head and neck region is the lingual [1] and sublingual location followed by the trachea, submandibular gland, lateral cervical region, maxilla, palatine tonsils, carotid bifurcation, iris of the eye, and pituitary gland. ETT may be also found in the cardiac muscle, ascending aorta, thymus, esophagus, duodenum, gall bladder, stomach bed, pancreas, mesentery of the small intestine, porta hepatis, adrenal gland, ovary (strumaovarii), fallopian tube, uterus, and vagina [2, 3]. Lingual thyroid accounts for 90% of ETT [2, 7, 8]. LT was first noticed by Hickmann in 1869 [5, 8–10]. The orthotopic thyroid gland is absent in 70% of cases with lingual thyroid [4, 7, 8, 10]. As in our case, thyroid was absent in a normal location. Hypothyroidism is present in 70% of LT patients [3, 4, 7]. Reported lingual thyroid is more among patients with thyroid disease [3]. LT causes hyperthyroidism rarely and also has malignant potential (1 in 300 cases) [2, 11]. Reported histotypes are papillary, follicular, mixed follicular and papillary, Hurthel cell, and medullary [2, 3]. Increased demand for thyroxine during puberty and pregnancy increases the work of the only functional ectopic tissue and, hence, increases LT size [2, 6, 8, 11]. This explains why the reported lingual thyroid is common among females. The amount of thyroid hormone secretion may not be enough during increasing hormonal demand could have contributed to increased LT size in our patient causing symptoms. Patients with enlarged LT present with dysphagia, dysphonia, foreign body sensation in the throat, cough, pain, bleeding, and dyspnea [4, 8, 11, 12].

Examination of the oral cavity to look for the nodule in the base of the tongue and palpation of the neck to assess the thyroid gland in orthotopic location is an essential initial evaluation [7, 11]. High-resolution ultrasonography (USG) is a readily available and noninvasive tool in the evaluation of the thyroid gland in the orthotopic location and detection of the ectopic thyroid nodule [6]. Color Doppler can aid to demonstrate the vascularity of the nodule. Computed tomography (CT) and magnetic resonance imaging (MRI) are valuable tools to define the location of the mass, presence of calcification, relation to the surrounding structure, and degree of luminal obstruction. A contrast-enhanced CT study is helpful to characterize the pattern of enhancement. The presence or absence of the orthotopic thyroid gland can also be defined by CT or MRI [3, 12]. A CT scan can also be helpful to detect possible nodules in mediastinum [2]. 99 m pertechnetate can localize ectopic thyroid tissue with a degree of activity and determine the presence of an orthotopic thyroid gland [3, 7, 11]. Fine-needle aspiration cytology (FNAC) helps to confirm the ectopic thyroid tissue and to rule out malignancy [2–4, 11].

Management of the lingual thyroid depends upon the severity of clinical symptoms and complications. Asymptomatic euthyroid patients need regular follow-up [3, 10]. Patients with subclinical hypothyroidism with symptoms or overt hypothyroidism are managed conservatively with exogenous thyroid hormone supplement to suppress and reduce the size of the ETT resolving the symptoms [4–11], as in our case. Patients with the only functional lingual thyroid tissue (LT) can be excised and autotransplanted in the muscles of the neck [3–6]. Ablative radioiodine therapy is considered in patients unfit for surgery or refusing intervention [4–9]. Surgical excision is indicated in symptomatic patients not responding to conservative management and patients with severe respiratory tract obstruction, bleeding, ulceration, and malignancy [3–10]. Surgical removal of the only functioning thyroid tissue needs lifelong hormone replacement therapy [8, 12].

## 4. Conclusions

Lingual thyroid is a rare clinical entity that presents as a nodule in the base of the tongue. Meticulous diagnostic workup including clinical examination, biochemical profile, imaging tools, radioisotope scanning, and histopathology is necessary to plan the management. Lingual thyroid with mild symptoms and subclinical hypothyroidism can be managed conservatively.

## Data Availability

The data supporting the results are available on request to the authors.
